# Protective Effects and Mechanism of Radix Polygalae Against Neurological Diseases as Well as Effective Substance

**DOI:** 10.3389/fpsyt.2021.688703

**Published:** 2021-12-17

**Authors:** Ning Jiang, Shanshan Wei, Yiwen Zhang, Wenlu He, Haiyue Pei, Hong Huang, Qiong Wang, Xinmin Liu

**Affiliations:** ^1^Sino-Portugal TCM International Cooperation Center, The Affiliated Traditional Chinese Medicine Hospital of Southwest Medical University, Luzhou, China; ^2^Research Center for Pharmacology and Toxicology, Institute of Medicinal Plant Development (IMPLAD), Chinese Academy of Medical Sciences and Peking Union Medical College, Beijing, China; ^3^Institute of Food Science and Technology, Chinese Academy of Agricultural Sciences (CAAS), Beijing, China

**Keywords:** neurological diseases, protective effects, review, animal, Radix Polygalae

## Abstract

Radix Polygalae (also known as Yuanzhi in China) is the dried rhizome of *Polygala tenuifolia* Willd. or *Polygala sibirica* L., which is a famous Chinese herb and has been widely used for centuries in traditional medicines including expectorants, tonics, tranquilizers, antipsychotic, and so on. This article reviews the neuroprotective effects of Radix Polygalae in preclinical models of central nervous system (CNS) disorders, especially anxiety, depression, declining cognition, Alzheimer's disease (AD), and Parkinson's disease (PD). The chemical composition of Radix Polygalae as well as the underlying mechanisms of action were also reviewed. We found that Radix Polygalae possesses a broad range of beneficial effects on the abovementioned conditions. The multifold mechanisms of action include several properties such as antioxidant and associated apoptotic effects; anti-inflammatory and associated apoptotic effects; neurogenesis, regeneration, differentiation, and neuronal plasticity improvement; hypothalamic–pituitary–adrenal axis (HPA) regulation; neurotransmitter release; and receptor activation (A_2A_R, NMDA-R, and GluR). Nevertheless, the detailed mechanisms underlying this array of pharmacological effects observed *in vitro* and *in vivo* still need further investigation to attain a coherent neuroprotective profile.

## Introduction

The central nervous system (CNS) is the most important regulatory system in the human body, playing a leading role in various life systems. With the continuous development of society and economy and the acceleration of industrialization and urbanization, the pressure of social competition is increasing and the pace of peoples' lives is also speeding up. Consequentially, there is an increasing prevalence of neurological disease, especially for mental disorders such as anxiety, depression, cognitive dysfunction, and neurological conditions such as Alzheimer's disease (AD) and Parkinson's disease (PD) (1, 2). According to the World Health Organization, the lifetime prevalence and 12-month prevalence rate of anxiety disorders worldwide were 5–25 and 3.3–20.4%, respectively, and major depression was listed as the third-highest cause of the global disease burden in 2008 and was predicted to take the lead by 2030 (3, 4). In 2015, the number of patients with AD reached 46.8 million worldwide, and by 2050, the number of patients with AD is expected to increase to 131.5 million. All of these mental disorders and neurodegenerative diseases cause great damage to the physical and mental health of patients.

In spite of its prevalence and high costs for the society, neurological disorders are often not correctly diagnosed and treated. Current drugs and methods used to prevent and manage neurological diseases are very limited (5), and treatments to slow, stop, or reverse the course of the disease constitute a great medical need. From 1998 to 2014, 123 AD drugs entered clinical trials, but only four of them were approved by the FDA, with a failure rate of 97%. Several medicinal plants and/or their active ingredients are widely used in the treatment of CNS diseases, showing high safety and remarkable therapeutic effects (6, 7). For example, panax notoginsenoside tablets can effectively treat insomnia and anxiety (8), and ginsenoside Rb1 has been developed as a potential therapeutic drug for AD (9, 10). Thus, it is urgent to explore natural active ingredients as sources of new therapy for neurological diseases.

Radix Polygalae (RP), extensively used as a folk medicine, is derived from the dried root of *Polygala tenuifolia* Willd. or *Polygala sibirica* L. Widely used in traditional Asian medicine in China, Japan, and South Korea for a long history, PR is considered as a top-grade herbal medicine that is described to enter the heart and kidney meridian in Shennong's Classic of Materia (*Shen Nong Ben Cao Jing*) and is also believed to be effective in soothing the nerves, dispelling phlegm, and dissipating edema. Among the chemical composition of RP, triterpenoid saponins, ketones, and oligosaccharide esters are thought to be the basis of RP CNS effects (11). The neuroprotectant properties of RP may result from improvement of cholinergic system and restriction of oxidative stress (free radical scavenging) (12).

To date, there is no systematic review of RP data in the context of neurological conditions and psychotic disorders. This paper reviews phytocompounds, therapeutic effects, and mechanisms of action, aiming to comprehensively summarize the existing research on RP related to the CNS and analyze its preventive potential. It is hoped that this overview will help to explain the results of clinical trials and promote the development of new treatments based on traditional knowledge in the area.

A literature search was conducted using the PubMed database. We searched for studies that were published from January 1, 1998, to December 30, 2020. The search strategy combined the following keywords and corresponding terms in the title and/or abstract: “Radix Polygalae,” “central nervous system disorders,” and “mental and psychotic disorders.”

## Chemical Composition

**Figure 1:**
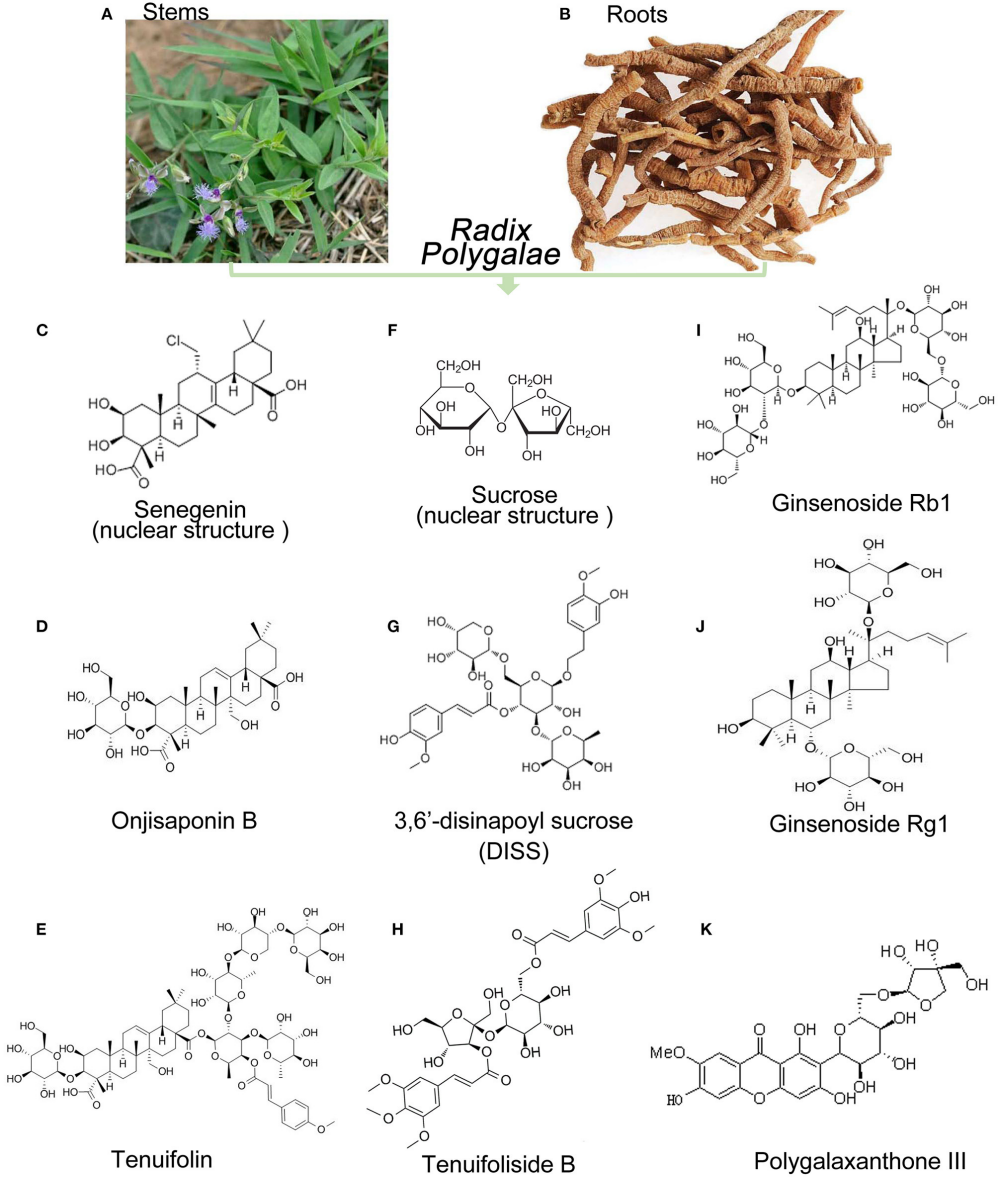
Sources and main chemical structure of the representative components of Radix Polygalae. **(A,B)** Stems and roots of RP; **(C)** senegenin as the mother nuclear structure of polygala saponins; **(D)** onjisaponin B; **(E)** tenuifolin; **(F)** sucrose as the mother nuclear structure of polygala saponins; **(G)** 3,6 ′-disinapoyl sucrose 3,6 ′ (DISS); **(H)** tenuifoliside B; **(I)** ginsenoside Rb1; **(J)** ginsenoside Rb1; **(K)** polygalaxanthone III.

The components of RP include saponins, oligosaccharides, ketones, alkaloids, polysaccharides, and flavonoids, and it has pharmacological effects related to sedation, anticonvulsive action, antidepressant action, anti-myocardial ischemic action, and learning and memory enhancement, as shown in Figure 1. Due to its neuroprotective effects on nerve cells, such as antidepressive effects and learning and memory improvements (12, 13), these extracts and active ingredients are receiving increasing attention from researchers.

### Polygala Saponins

The polygala saponins, as the active ingredients, are rich in the roots and stalks of RP (Figures 1A,B). The total saponin content of *P. tenuifolia* Willd. in the aerial parts (stem and leaf) is up to 2.46% and that in root is 3.29%. The total saponin content in the stems and leaves of *P. sibirica* L. is 1.50 and 1.61%, respectively, and ginsenosides and saponins are mainly in the roots. However, there are differences in the structure of ginsenosides and polygalasaponin (senegenin): the basic nucleus of the polygala saponin is an oleanolic acid-type pentacyclic triterpenoid, and the two types are the dammarane-type tetracyclic triterpenoid saponin and the oleanolic acid-type pentacyclic triterpenoid saponin, respectively (14), such as the protopanaxadiol saponin Rb1 (15, 16) and the original ginseng triol-type saponin Rg1 (17, 18) (Figures 1I,J). Senegenin (Figure 1C) is treated as the mother nucleus of the polygala saponins and can be linked to different sites, including sugars (12, 17, 19, 20) such as onjisaponin B (Figure 1D) and tenuifolin (Figure 1E), as reported in the previous studies (21, 22).

### Oligosaccharide Esters

Polygala oligosaccharide ester is a unique chemical component found in *Polygala* species and is mainly concentrated in the roots (23). Its mother nucleus is mainly composed of glucose, sucrose, or other trisaccharide (Figure 1F) that are linked to glucose or rhamnose with glycosidic bonds to form oligosaccharides and further combined with organic acid components (acetic acid, benzoic acid, and cinnamic acid) to form an ester, in which the oligosaccharide (no more than five molecular sugars) is glycosidically bonded (24, 25). Currently, hundreds of oligosaccharide esters have been isolated from RP, such as tenuifoliose A Q (Supplementary Material 1) and tenuifoliside A E (as shown in Figure 1G). However, 3,6′-disinapoylsucrose (DISS) (Figure 1H) is unique to the genus *Polygala* (21, 22) and *Polygala* oligosaccharide (12, 17, 19, 22).

### Polygalaxanthone

Polygalactones are a class of phenolic compounds in the form of derivatives, represented by polygalaxanthone III (21, 22, 26) (Figure 1K), that have diuretic, antibacterial, anticancer, and antidepressant among other biological activities. In addition, a variety of alkaloids were isolated from Polygala: N-formyl halo, 1-butoxycarbonyl-β-carboline, 1-ethoxycarbonyl-β-carboline, and 1-methoxycarbonyl-β-porphyrin. However, the pharmacological action of alkaloids on the CNS has been reported to be low.

At the same time, less has been reported on chemical modifications of the active natural components to further enhance the effects in the treatment of antipsychotic diseases, which should be the focus of scientific research. Based on existing natural compounds with good activity, chemical modifications further carry out targeted structural transformation, improve binding sites, improve solubility, and improve tissue targeting, which provide excellent prospects for psychotropic drug development.

## Biology

### Sedative and Anxiolytic Effects

Recent studies report anxiolytic-like and anticonvulsant effects obtained with the RP extract and/or its active ingredients (27–29). Table 1 summarizes the effects in anxiety-like behavior, levels of monoamines, and quality of sleep time by *P. tenuifolia* (a major plant source of RP) root extract, polygalasaponins, and tenuifolin in experimental models. However, the potential mechanisms of its anxiolytic-like and anticonvulsant effects remain unclear.

**Table 1:** Summarized anxiolytic and antidepressant effects of RP in the nervous system.

**Models**	**Methods**	**Drug material**	**Effects**	**Mechanisms**	**RF**
Aged mice; SH-SY5Y cells	TST and FST	Ethanol extracts Four fractions	↑ Anti-immobility-like effects; ↑ Cell proliferation; ↓ SH-SY5Y injury	↑ Cell proliferation; Antidepressant-like effects	(30)
CUMS, mice	TUNEL, FST, TST, FUST, and LHP	Polygalae extract	↓ Behavioral despair; ↓ NMDA neurotoxicity; Hedonic-like behavior; ↓ Number of failed escapes	↑ BDNF; ↓ GluR1 phosphorylation; ↑ Glutamatergic synapses	(31)
CUMS, mice	FST and TST	Extract	↑ antidepressant-like activity	↓ Triple monoamine reuptake	(32)
CMS Rats	SPT	DISS	↑ SOD activity; ↓ cortisol, MAO-A, and MAO-B; ↓ Lipid peroxidation and MDA	MAO, HPA axis Oxidative systems	(33)
CRS Rats	SPT, FST, and NSFT	Extract	↑ Antidepressant-like activity; ↑ LC3-II and beclin1; ↑ NLRP3, ASC, and caspase-1; ↑ Pro-inflammatory cytokines; ↓ the level of p62; Activated microglia and impaired astrocyte	AMPK-mTOR pathway Inhibit neuroinflammation	(24)
CMS, rats	SPT, FST, and TST	DISS	↑ Reward reaction; ↑ sucrose consumption; ↓ Serum CORT, ACTH, and CRH	HPA axis; ↑ Glucocorticoid receptor; ↑ Mineralocorticoid receptor	(34)
Mice	EPM	Tenuifolin	↑ total sleep time; ↑ bouts of episodes; ↑ C-Fos positive ratios; ↓ NA; ↑ GABA, Ach	CREB-BDNF; ERK1/2 and CaMKII	(27)
Mice	EPM, OFT, and hypnosis tests	Saponins	↑ Central crossing counts; ↑ central/total ambulation; ↓ Number of rearings;↑ Head-dips; ↑ sleep duration; ↓ sleep latency	Evident anxiolytic and sedative-hypnotic activities	(28)
Cocaine; Mice	Locomotor activity and CPP	Extracts	↓ Hyperactivity; ↓ Locomotor activity	↑ Adenosine A_2A_ receptor	(29)

*TST, tail suspension test; FST, forced swimming test; SPT, sucrose preference test; FUST, female urine sniffing test; LHP, learned helplessness paradigm; NMDA, N-methyl-D-aspartate; BDNF, brain-derived neurotrophic factor; DISS, 3,6′-disinapoyl sucrose; CMS and CUMS, chronic unpredictable mild stress; CRS, chronic restraint stress; HPA, hypothalamic–pituitary–adrenal axis; EPM, elevated plus maze; NA, noradrenaline; Ach, acetylcholine; OFT, open field test; CPP, conditioned place preference; RF, reference. Other abbreviations are displayed in the literature*.

The extracted polygalasaponins possess evident anxiolytic and sedative-hypnotic activities, with a relatively safe dose range (28). In the open field test, the administration of polygalasaponins (40–160 mg/kg) significantly increased central crossing and the percentage of central/total ambulation and markedly inhibited the number of rearing and defecations. Additionally, increases in the number of head dips in the board hole and the time spent in the open arms of the elevated plus-maze test, prolonged sleep duration, and shortened sleep latency were observed (28).

Tenuifolin administration (40 and 80 mg/kg, *p.o*.) significantly prolonged total sleep time, increased the amount of non-rapid eye movement and rapid eye movement sleep, and bouts of episodes. Neurochemical correlations include increased c-Fos positive ratios; reduced noradrenaline levels in the locus coeruleus (LC), ventrolateral preoptic area (VLPO), pontomesencephalic tegmental area (PPT), and laterodorsal tegmental area (LDT); elevated GABA levels in the VLPO and LC; and increased acetylcholine (Ach) levels in the LDT and PPT (27). Overall, the data supports the use of *P. tenuifolia* root as anxiolytic and hypno-sedative drug in traditional medicine.

*P. tenuifolia* root extract contains the adenosine A_1_ receptor antagonists 8-cyclopentyl-1,3-dimethylxanthine and alloxazine, which are related to the effect of PTE in preventing cocaine-induced behavioral effects (29).

### Antidepressant Effects

RP is thought to exert a variety of antidepressant effects. As shown in Table 1, various extracts and purified fractions of RP showed significant antidepressant-like effects in animal models. The mechanism of action includes the following: improving neural cell by reducing serum CORT, ACTH, and CRH levels; increasing SOD activity; decreasing brain monoamine oxidase (MAO-A and MAO-B) activity and serum malondialdehyde (MDA) levels; promoting autophagy and inhibiting neuroinflammation; regulating the glutamate AMPA receptor, and decreasing GluR1 phosphorylation. The studies were mainly about the four fractions of an ethanol extract as well as the active components DISS and tenuifoliside A (TEA).

The oral administration of RP (200 mg/kg) showed significant anti-immobility effects on the tail suspension test (TST) and forced swim test (FST), inhibited corticosterone-induced SH-SY5Y cell injury, improved cell viability, and promoted cell proliferation (30). The extracts had antidepressant-like activity *via* inhibition of triple monoamine reuptake (32). Bioassay-guided screening methods showed two bioactive compounds: DISS and TEA (33, 34).

Oral administration of 0.1 mg/kg RP extract decreased behavioral despair in the forced swim and tail suspension tasks, increased hedonic-like behavior in the female urine sniffing test, and decreased the number of failed escapes in the learned helplessness paradigm in male C57Bl/6 mice (31). This study suggested that RP exerted rapid-onset antidepressant effects by regulating the AMPA receptor and decreasing GluR1 phosphorylation at serine-845 in the hippocampus, thus modulating glutamatergic synapses in critical brain circuits of depression. Moreover, Wistar rats were exposed to 6-h restraint stress daily for 28 days, and RP extract (0.5 and 1 g/kg) exerted a remarkable antidepressant activity in behavioral despair mice and induced rats, probably by promoting autophagy and inhibiting neuroinflammation (24).

DISS, an oligosaccharide ester component isolated from the roots of *P. tenuifolia*, improved the reward reaction and increased sucrose consumption in rats (33, 34). In addition, DISS (10–20 mg/kg, gavage) remarkably reduced serum CORT, ACTH, and CRH levels; increased SOD activity in the plasma and brain; decreased brain MAO-A and MAO-B activity in the brain; reduced plasma cortisol levels (34); and downregulated MDA levels in the serum and brain (33), suggesting that the antidepressant-like effects of DISS might be related to modulating the hypothalamic–pituitary–adrenal (HPA) axis and enhance the expression of glucocorticoid and mineralocorticoid receptors in CMS rats (33, 34).

### Learning and Memory Improvements

Learning and memory disorders arise from distinct age-associated processes, and aging animals are often used as a model of memory impairment. As the memory-improving effects of RP have been reported in various animal models, it is more and more commonly used in some Asian countries for this purpose.

As it can be seen in Table 2, multiple extracts and/or the active ingredients of *P. tenuifolia* extract are active in memory-related animal models. In a postoperative cognitive dysfunction model in elderly rats having undergone splenectomy, *P. tenuifolia* crude extract (35), a precipitate fraction (PTB) from RP (37), and senegenin can significantly inhibit the mRNA and protein expression levels of several key pro-inflammatory cytokines, reducing tumor necrosis factor-α (TNF-α), interleukin-1β (IL-1β), IL-6, and IL-8 levels, in the hippocampal tissues (41). In addition, a saponin-rich fraction obtained by the purification of *P. tenuifolia* crude extract significantly improved learning and memory in normally aged mice in the Morris water maze (MWM), the step-down passive avoidance tests (35), and the eight-arm radial maze task in rats (37) and in 5xFAD transgenic mice (36). All of these effects may be involved in the regulation of the TLR4/MyD88/NF-κB and TLR4/TRIF/NF-κB signaling pathways.

**Table 2:** Summarized effects of RP on improving learning, memory, and cognitive function in the nervous system.

**Models**	**Methods**	**Drug material**	**Effects**	**Mechanisms**	**RF**
Aged mice	MWM SPAT	Crude extract	↑ SOD-2; ↑ Impaired spatial memory; ↑ SOD and CAT activities; ↓ MAO and AChE activities	↓ MAO and AChE Antioxidant properties	(35)
Neurons Aβ and H_2_O_2_; AD model mice	NOR; TUNEL; DCF-DA assay	PSM-04	↓ Cognitive impairments; ↓ Amyloid plaques and gliosis; ↓ Cognitive impairment	↓ Oxidative stress ↓ Apoptosis	(36)
Rats Scopolamine	ERM; RMP	Extracts, PTB PTBM, and SNPA	↓ number of total errors; ↑ Short-term memory	↑ memory	(37)
Mice Scopolamine	MWM	P A	↑ Escape latency time; ↑ SOD, ↓ MDA, glutathione; ↑ Number of crossings	↓ AChE activity; ↑ ChAT; Ach; ↓ Neuroinflammation	(38)
SH-SY5Y; Mice; rats; Primary neurons	MWM and SPAT	Triterpenoid saponins	↑ Synaptic transmission; ↑ Cognition and memory activity; ↑ BDNF, NGF; ↓ toxicity; ↑ ChAT	MAPK; CREB-BDNF; ERK1/2 and CaMKII	(39)
Mice Aβ25–35;	MWM and SPAT	Tenuifolin and fallaxsaponin A	↓ Cognitive deficits; ↓ apoptosis in PC12 cells	↑ COGNITIVE function	(40)
POCD rats; Splenectomy	MWM	Senegenin	↓ TLR4 ↓ Cognitive impairment; ↓ TNF-α, IL-1β, IL-6, and IL-8; ↓ Key pro-inflammatory cytokines;	↓TLR4/TRIF/NF-κB; ↓TLR4/MyD88/NF-κB; ↓NF-κB phosphorylation	(41)
APP695, SPA4CT	*In vitro* SH-SY5Y	Tenuigenin	↓ Aβ and C99; ↓ BACE1proteolytic activities	↓ BACE1; ↓ Secretion of Aβ	(42)
Potassium cyanide scopolamine impairment rats	SPAT	Tenuifoliside B	↑ Cerebral protective effect; ↑ Oxotremorine-induced tremors; ↓ Impairment of performance	↑ Cholinergic system	(43)

*MWM, Morris water maze; SPAT, step-down passive avoidance tests; CAT, catalase; NOR, novel object recognition; SOD-2, superoxide dismutase-2; ERM, eight-arm radial maze; PA, polygalacic acid; AChE, acetylcholinesterase; ChAT, choline acetyltransferase; Ach, acetylcholine; TNF-α, tumor necrosis factor-alpha; IL-1β, interleukin-1β; C99, C-terminal 99 amino acids of APP; MAPK, mitogen-activated protein kinase*.

Reports proved that the crude extract of *P. tenuifolia* (100 and 200 mg/kg) significantly improved impaired spatial memory in aging mice in the MWM and step-down tests, an effect that was related to the disruption of superoxide dismutase (SOD) and catalase (CAT) activities, the inhibition of MAO and acetylcholinesterase (AChE) activities, and the decrease in the levels of MDA in the brain tissue (35) (see Table 2). In addition, the oral administration of PTB of *P. tenuifolia* root and a saponin-rich fraction obtained from PTB (100 and 200 mg/kg) decreased the number of total errors of working memory as evaluated in the rat eight-arm radial maze tasks (37). Additionally, *P. tenuifolia* extracts had beneficial effects on the scopolamine-induced dysfunction of the cholinergic system-induced memory impairment in the radial maze performance (37).

The oral administration of RP (3, 6, and 12 mg/kg; 14 days) reversed scopolamine-induced amnesic effects such as increased escape latency time, decreased number of crossings, and shortened time spent in the target quadrant. RP significantly improved the cholinergic system reactivity by decreasing AChE activity, increasing choline acetyltransferase (ChAT) activity, and elevating levels of ACh, which ameliorated neuroinflammation and inhibited oxidative stress in mice (38).

Finally, senegenin, an active component of extracts from *P. tenuifolia* root, significantly inhibited the mRNA and protein expression of several key pro-inflammatory cytokines by downregulating the inflammation signaling pathways dependent on TLR4/MyD88/NF-κB and TLR4/TRIF/NF-κB and by reducing TNF-α, IL-1β, IL-6, and IL-8 levels in hippocampal tissues in the postoperative cognitive dysfunction model of elderly rats having undergone splenectomy (41). These results suggest that RP might exert a significant neuroprotective effect by improving learning and memory, driven in part by the modulation of cholinergic activity and neuroinflammation.

### Alzheimer's Disease

AD is a progressive degenerative disease of the CNS that involves impaired language and memory function. The accumulation of the amyloid β-protein (Aβ) is a pivotal pathological factor in AD. Most studies and searches for effective drugs have been conducted from the perspectives of cholinergic system decline, neuronal apoptosis, oxidative stress, and neuro-inflammation induced by the disease.

As mentioned, RP has been widely used in neurodegenerative diseases in traditional Chinese medicine. As summarized at Table 2, our review shows that RP can alleviate pathological changes in mice, reduce neurofibrillary tangles and amyloid fibrosis senile plaques in the hippocampus and cerebral cortex, and promote the regeneration of new neurons in the dentate granule cells (36, 39, 40, 42). The authors suggest that these effects may be associated with RP antioxidative, anti-inflammatory, and anti-apoptotic effects; HPA axis regulation; increase in monoamine neurotransmitter release; BDNF and NGF expression; neuronal synaptic plasticity; and nerve cell proliferation. In addition, RP extracts and its active ingredients (tenuifolin and fallaxsaponin A) may decrease Aβ secretion *via* BACE1 (beta-secretase) inhibition.

PSM-04, an extract of RP, exhibited significant neuroprotective effects against neurotoxicity induced by L-glutamate or oligomeric Aβ, decreased oxidative stress induced by H_2_O_2_, and decreased apoptotic cell death induced by oligomeric Aβ in primary cortical neurons (36); in addition, PSM-04 significantly alleviated cognitive impairments in 5xFAD transgenic mice by increasing superoxide dismutase-2 (SOD-2) protein levels, alleviating cognitive impairment, and decreasing amyloid plaque deposition (36).

Tenuifoliside B showed cerebral protective effects against potassium cyanide-induced anoxia in mice and had an ameliorative effect on scopolamine-induced impairments in the passive avoidance task in rats as well as an ameliorative effect on the reduction in cholinergic function on rat models induced by Aβ (43). Studies on the mechanism of action found that tenuifolin B could inhibit secretion of Aβ in neuroblastoma cells stably transfected with two amyloid precursor protein (APP) constructs: the APP695 cDNA (SH-SY5Y APP695) and the C-terminal 99 amino acid residues of APP plus the signal peptide (SH-SY5Y SPA4CT). Tenuigenin inhibited the secretion of Aβ and the C-terminal 99 amino acids of APP (C99) in SH-SY5Y APP695 cells, but did not change the Aβ and C99 levels in SH-SY5Y SPA4CT cells. Fluorescence resonance energy transfer (FRET) assays showed that tenuigenin inhibited the proteolytic activities of BACE1 on its substrate *in vitro* (42).

Furthermore, tenuifolin, which is isolated from *P. tenuifolia* roots, showed neuroprotective effects in Aβ_25−35_ damage-induced PC12 cells *in vitro* and significantly alleviated cognitive deficits induced by the intrahippocampal injection of Aβ_25−35_ in mice and APP/PS1 transgenic AD mice model. Its extracts and active ingredients can reduce cell apoptosis by ameliorating neuro-inflammation and oxidative stress and can inhibit cell toxicity by regulating BDNF expression, by upstream ERK1/2 pathway, CaMKII activation, and cyclic AMP-responsive element-binding protein (CREB)-mediated BDNF transcription (40, 44).

Taken together, these results suggest that more studies are needed in the context of RP products as anti-AD medication.

### Parkinson's Disease

Based on the reported biological and pharmacological activities of *P. tenuifolia*, it can be argued whether it could be useful in the prevention and/or treatment of PD that share with AD aspects such as neuroinflammation. In fact, it was shown in *in vitro* and *in vivo* PD models of the BV2 microglia cells and the MPTP-induced mice model that tenuigenin significantly increased striatal dopaminergic levels and improved motor impairment induced by MPTP, ameliorated the degeneration of dopaminergic neurons, and inhibited NLRP3 inflammasome activation in the substantia nigra (45). Tenuigenin reduced the production of intracellular reactive oxygen species (ROS), the subsequent caspase-1 cleavage, and IL-1β secretion in BV2 microglia cells and attenuated microglia activation induced by lipopolysaccharide (LPS) *via* suppressing NLRP3 inflammasome (45).

PR extracts have protective effects on dopaminergic neurons *via* their antioxidant and anti-apoptotic activities (46). PR extracts inhibited 6-OHDA-induced PC12 cell damage, alleviated caspase-3 activity and the production of ROS and NO, and protected mesencephalic dopaminergic neurons from MPP (+)-induced toxicity. It also protected dopaminergic neurons and fibers from MPTP-induced toxicity in the substantia nigra pars compacta (SNpc) and TH-IR fibers in the striatum (ST) (47). These results suggest that RP extracts and tenuigenin may be useful in PD therapy by counteracting the inflammatory response, oxidative stress, and associated apoptosis.

**Figure 2:**
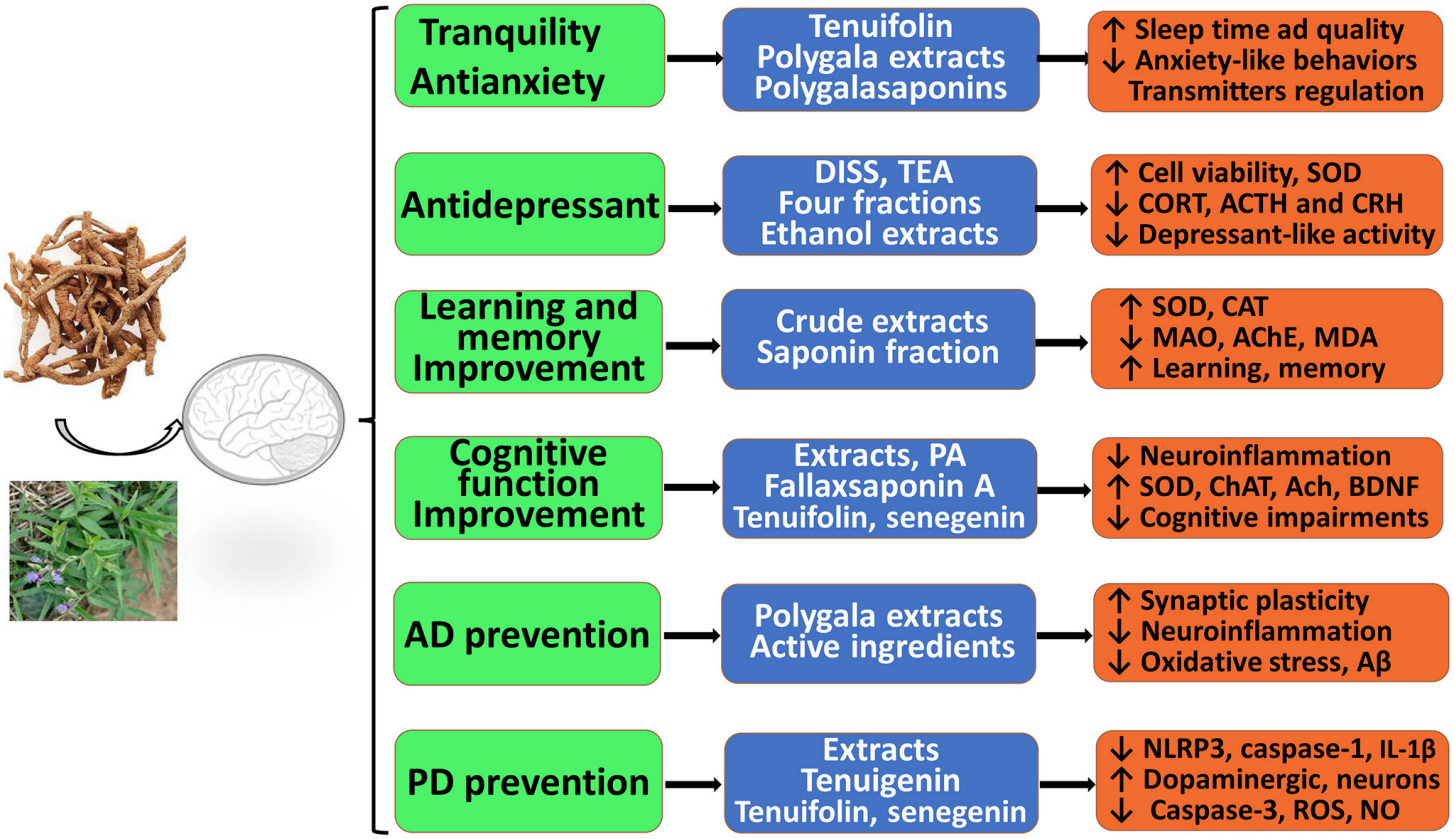
Therapeutic and preventive actions of RP against central nervous system (CNS) neurological and psychotic diseases *in vitro* and *in vivo*. It possesses significant pharmacological activities and protective effects with administration of therapeutic material basis, including RP extracts, multiple effective fractions, and single components.

As described above, RP extracts, multiple fractions, and various effective components have significant neuroprotective effects against CNS neurological and psychotic diseases *in vitro* and *in vivo*. As shown in Figure 2, these extracts, active fractions, and active ingredients, including saponins, ketones, oligosaccharides, and alkaloids, have significant pharmacological activities and protective effects against anxiety, depression, cognitive decline, AD, and PD, which suggests that RP may provide effective tools to combat AD, PD, depression, and other neurological conditions or disorders. Thus, RP has been attracting more attention from researchers. First, RP extracts, polygalasaponins and tenuifolin, may significantly alleviate anxiety-like behavior, decrease locomotor activity, regulate levels of monoamine neurotransmitters, and improve sleep time and quality; thus, RP has evident tranquility and anti-anxiety effects. However, the potential mechanisms of anxiolytic-like and anticonvulsant effects have been less frequently reported and remain unclear. Second, the various PR extracts, purified fractions, and several active ingredients (DISS, TEA, etc.) may exert obvious antidepressant effects by inhibiting depression-like behaviors, improving neural cell viability and proliferation, reducing oxidation and apoptosis, and modulating glutamatergic synapses and the HPA axis. Third, RP extracts and their active ingredients (polygalacic acid, tenuifoliside B, tenuifolin, fallax saponin A, and senegenin) may improve learning, memory, and cognitive functions; alleviate cognitive impairments; ameliorate neuroinflammation; inhibit oxidative stress; and reduce Aβ secretion and neuronal apoptosis. These findings show the excellent prospects of RP for treating AD. In addition, RP shows promising clinical use for PD therapy.

## Neuroprotective Mechanisms

### Neuronal Synaptic Plasticity and Proliferation

It is well-known that neuron density and function, neurotransmitter levels, and BDNF expression levels are decreased in AD patients, mostly replicated in animals subjected to experimental models of depression. The expression of BDNF has also been implicated in the effect of many neurological disorders, which is also involved in the expression of neuronal plasticity genes, so it is now also believed that neurodegenerative diseases are frequently associated with a loss of synapses and neurons (6). *P. tenuifolia* root extracts and their active ingredients proved to be neuroprotective against psychotic or neurodegenerative disease models by regulating the HPA axis, regulating neurotransmitter release, increasing BDNF and NGF expression, improving neuronal synaptic plasticity, and promoting nerve cell proliferation (Table 3).

**Table 3:** Neuroprotective mechanisms of Radix Polygalae against oxidation stress, inflammatory, and apoptosis in the nervous system.

**Models**	**Methods**	**Drug material**	**Effects**	**Mechanisms**	**RF**
Glu SY5Y cells	*In vitro*, inhibitor U0126	DISS TEA	↓ cell viability; ↑ CRTC1 and BDNF; ↓ NOS hyperactivation; ↑ CREB phosphorylation	PI3K; MAPK/ERK1/2; CREB-BDNF	(48)
Rats Brain slices	*In vitro*	Tenuifoliside	↑ Slope of fEPSPs; ↓ Paired-pulse facilitation ratio; ↑ Frequency and amplitude	↑ Synaptic; ↓ Intracellular calcium	(49)
Rat cortical neurons	*In vitro*	Senegenin	↑ Akt phosphorylation; ↑ Neuronal outgrowth and survival	PI3K/Akt	(50)
Rat Glioma C6 cells	*In vitro*; inhibitors U0126 and LY294002	TEA	↓ apoptosis; ↑ cell viability and BDNF; ↑ ERK and Akt phosphorylation	BDNF/TrkB; ERK/PI3K-CREB	(51)
H_2_O_2_ Glutamate, SY5Ycell	*In vitro*; inhibitors U0126, KN93, or K252a	DISS	↑ BDNF; ↑ cell viability; ↑ CREB phosphorylation	CREB-BDNF; ERK1/2 and CaMKII	(52)
Rat neurons	*In vitro*	Root extract	↑ proliferation; ↑ Neurite outgrowth	Neurogenesis	(53)
Rat neurons	*In vitro*; NMDA	Extract	↓ (Ca^2+^)I and ROS; ↓ Cell death and glutamate release	↓ Oxidation stress; ↓ apoptosis	(54)
LPS astrocytes	*In vitro*	DISS and TEA	↓ TNF-α and IL-1β	Anti-inflammatory activity	(55)
LPS, Macrophages, RAW264.7 cells	*In vitro*	TEA	↓ TNF-α and IL-1β; ↓ NO, iNOS, PGE2, and COX-2; ↓ Pro-inflammatory cytokines	↓ p-JNK; ↓ NF-κB; ↓ MAPK pathways	(56)
LPS, RAW 264.7 cells	*In vitro*	Tenuigenin	↓ JNK1/2, ERK1/2, and p38; ↓ IκBα phosphorylation; ↓ PGE2, NO, iNOS, and COX-2	MAPK, NF-κB, anti-inflammatory Nrf2/HO-1, and anti-oxidation	(57)
MPTP, Mice, BV2 cells	*In vitro*	Water extracts	↑ Striatal dopaminergic levels;dopaminergic neuron ↑ Dopaminergic neuron; ↓ ROS, caspase-1, and IL-1β;	↓ NLRP3 inflammasome activation	(45)
6-OHDA, PC12 cells; PD mice	*In vitro* and *in vivo*	Polygalae extracts	↓ ROS and NO ↓ cell damage and toxicity; ↓ Caspase-3	Anti-oxidant and anti-apoptotic activities	(46)
LPS, BV2 cells	*In vitro*	Water extract	↓ NO and PGE2; ↓ iNOS and COX-2;dopaminergic neuron ↓ Pro-inflammatory cytokines	↓ NF-κB; ↓ IkappaB-α; ↓ TLR4 and Myd-88	(58)

*ROS, reactive oxygen species; LPS, lipopolysaccharide; (Ca^2+^)_I_, intracellular Ca^2+^ concentration; TEA, tenuifoliside A; NO, nitric oxide; TNF-α, tumor necrosis factor-alpha; IL, interleukin-1β; iNOS, inducible nitric oxide synthase; PGE2, prostaglandin E2; COX-2, cyclooxygenase-2; p-JNK, p-c-Jun N-terminal kinase; MPTP, 1-methyl-4-phenyl-1,2,3,6-tetrahydropyridine; 6-OHDA, 6-hydroxydopamine; IκB-α, IkappaB-α*.

DISS and tenuifoliside (A, B, and C), two natural oligosaccharide esters from *P. tenuifolia* roots, improved neuronal synaptic plasticity, promoted nerve cell proliferation, and showed neuroprotective effects *via* inhibiting glucocorticoid-induced NOS hyper-activation, upregulating the phosphorylation of CREB, and increasing the expression of BDNF in corticosterone-induced SH-SY5Y cells (19). Senegenin was also found to promote neurite outgrowth and neuronal survival in primary cultured rat cortical neurons (50, 59). The studies showed that the effects may be related to stimulating different upstream pathways of CREB *via* the MAPK/ERK1/2, phosphoinositide-3 kinase (PI3K), and CREB-BDNF pathways, which was demonstrated *in vitro* (19). The neurotrophic effects of senegenin were significantly inhibited by the A_2A_ receptor antagonist ZM241385 and the specific PI3K inhibitor LY294002, suggesting that senegenin may be involved in the PI3K/Akt signaling pathway (50, 59).

Neuroprotective and anti-apoptotic effects were shown for TEA. TEA increased the levels of phospho-ERK and phospho-Akt, improved CREB phosphorylation at Ser133, and, thus, enhanced the release of BDNF in rat glioma cells C6. Inversely, these effects were blocked by ERK and PI3K inhibitors (U0126 and LY294002, respectively). Therefore, it was speculated that the neuroprotective effects of TEA in C6 glioma cells might be mediated by the BDNF/TrkB-ERK/PI3K-CREB signaling pathway (51).

RP root extract on proliferation of neural stem cells in the hippocampal CA1 region (2 mg/kg/day, 14 intraperitoneal injections) could increase the incorporation of bromodeoxyuridine (BrdU) into cells in the hippocampal CA1 region and promote the proliferation of neural stem cells in the rat hippocampus, an effect potentially beneficial to conditions like insomnia, neurosis, and dementia (53).

Tenuifoliside enhanced basic synaptic transmission by stimulating presynaptic intracellular calcium as shown by *in vitro* field potential electrophysiology and whole-cell patch clamp techniques (49). Tenuifoliside significantly enhanced the slope of field excitatory postsynaptic potentials (fEPSPs), reduced the ratio of paired-pulse facilitation, increased the frequency of spontaneous excitatory postsynaptic currents (mEPSCs), and increased their amplitude; these effects were blocked by chelating intracellular calcium with BAPTA-AM (49).

### Antioxidative Effects

RP extracts and their active ingredients show obvious neuroprotective effects against oxidative stress and apoptosis (60), while also improving nerve growth, neuronal plasticity, neurotransmitter reuptake, and neurogenesis.

Two oligosaccharide esters from RP, tenuifoliside and DISS, were shown to inhibit NOS hyper-activation, toxicity, and apoptosis in glutamate- and H_2_O_2_-damaged SH-SY5Y, ameliorating neuron viability in a dose-dependent manner. Both esters increased BDNF expression and CREB phosphorylation *via* the regulation of upstream MAPK/ERK1/2 and PI3K in the CREB-BDNF pathway (48). In *in vivo* rat models (Table 3), DISS showed distinct neuroprotective effects *via* the modulation of BDNF and CREB expression, and CREB-BDNF regulation was associated with the upstream activation of ERK1/2 and CaMKII, verified by the inhibitors (H89, LY294002, U0126, KN93, and K252a) (52).

In primarily cultured rat cerebellar granule neurons, RP extracts inhibited NMDA (1 mM)-induced neuronal cell death, inhibited glutamate release into the medium, inhibited the elevation of intracellular Ca^2+^ concentration [(Ca^2+^)i] (49), and inhibited the generation of ROS (54). It also inhibited 6-OHDA-induced PC12 cell damage and increased caspase-3 activity and the production of ROS and NO (46). In LPS-induced RAW 264.7 macrophages, RP dramatically upregulated heme oxygenase (HO)-1 and Nrf2 expressions, which were partially reversed by HO-1-siRNA and HO-1 inhibitors (57), suggesting that RP extracts have significant neuroprotective actions *via* its antioxidant and anti-apoptotic activities.

### Anti-inflammatory Effect

There is an increasing evidence suggesting that inflammation plays a crucial role in the development of psychotic diseases (61) (Table 3). Inflammation is triggered by various internal and external stress stimuli, which in turn can cause abnormal changes in the normal physiological functions of serotonin (5-HT) and the HPA axis, eventually leading to anxiety and depression. Studies have shown that *P. tenuifolia* root extract has obvious anti-inflammatory activity, which can improve cytokine levels, regulate inflammation-related pathways, and, thus, may be useful for treating psychosis (38, 45, 56, 58).

In primary cultures of mouse astrocytes, the RP aqueous extract inhibits TNF-α secretion and decreases IL-1β secretion. These results suggest that PTAE has anti-inflammatory activity in the CNS, assisting to counteract the neuroinflammation that accompanies various pathological conditions (55, 60).

Increasing evidence suggests that tenuigenin possesses antioxidative and anti-inflammatory activities (Table 3). Tenuigenin dramatically reduces prostaglandin E2 and NO production (57) and suppresses the production of pro-inflammatory cytokines such as TNF-α and IL-1β in LPS-induced RAW264.7 macrophages (56), which was related to decreased iNOS and COX-2 gene expression. Furthermore, PR extracts and TEA inhibited JNK1/2, ERK1/2, p38, and NF-κB (p65) phosphorylation and blocked IκBα phosphorylation and degradation. Together, tenuigenin showed obvious anti-inflammatory activity *via* the inhibition of the NF-κB and MAPK pathways (56, 57). Tenuigenin significantly increased striatal dopaminergic levels, improved motor impairment induced by MPTP, ameliorated the degeneration of dopaminergic neurons, and inhibited NLRP3 inflammasome activation in MPTP mice (45). Additionally, tenuigenin obviously reduced intracellular ROS production; suppressed NLRP3 inflammasome activation, subsequent caspase-1 cleavage, and IL-1β secretion; and attenuated microglia activation *via* suppressing the NLRP3 inflammasome in BV2 microglia cells (45). All the results suggest that the neuroprotective effects of tenuigenin are associated with the inhibition of NLRP3 inflammasome activation (45).

## Conclusions

**Figure 3:**
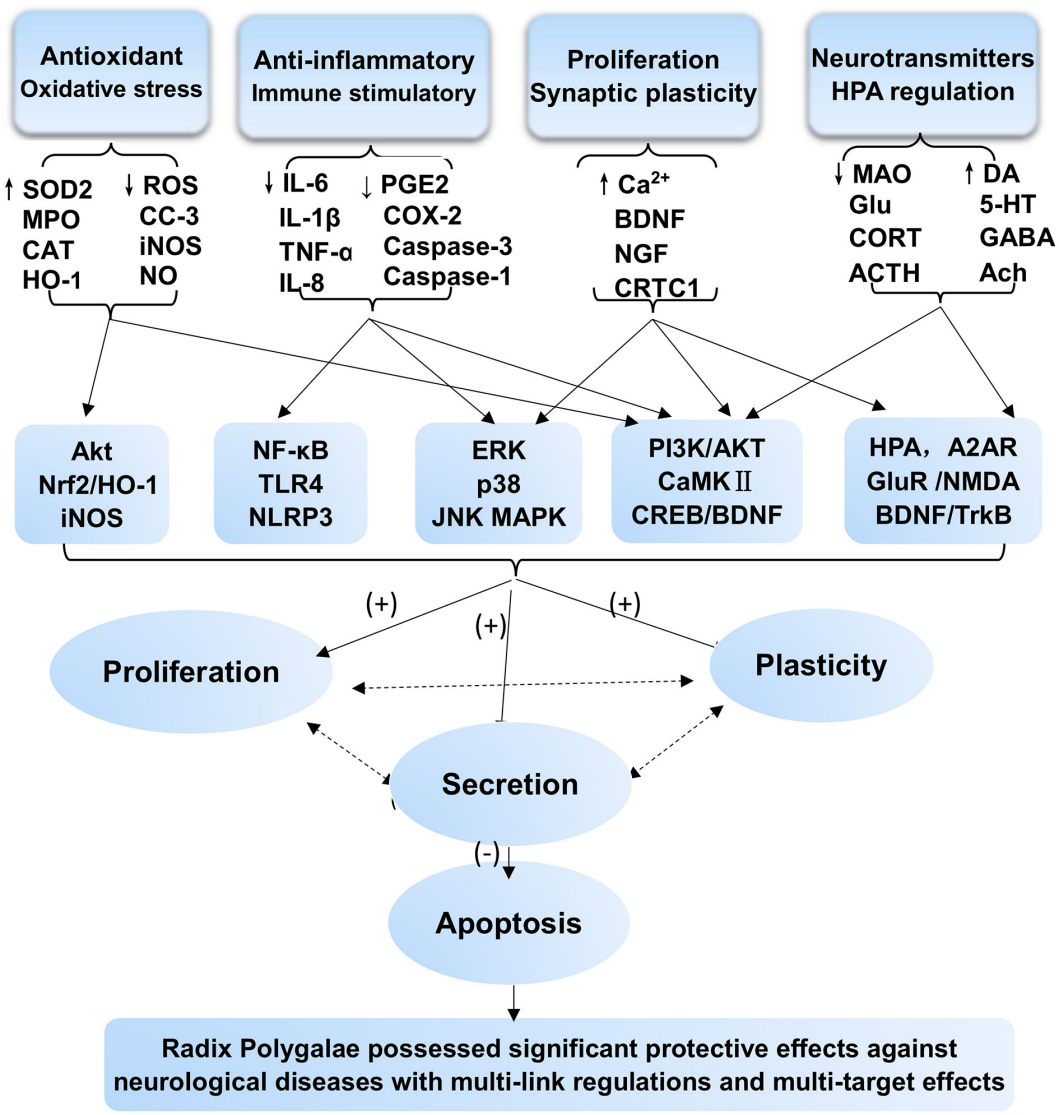
Protective effects and mechanism network analysis of RP against CNS neurological and psychotic diseases *in vitro* and *in vivo* with multiple links across regulatory mechanisms and multitarget effects. Minus sign (–) stands for downregulation or inhibition; plus sign (+) stands for upregulation or activation; question mark (?) stands for uncertainty or no determination.

RP, as a medicinal herb, is the dried root of *P. tenuifolia* Willd. or *P. sibirica* L. and has been widely used as an expectorant, tonic, tranquilizer, and antipsychotic agent. Its pharmacological effects are well-documented in extracts, extract fractions, and isolated compounds affecting various aspects of neurodegenerative disorders, including AD and PD, as well as neuropsychiatric disorders. This review summarized that RP has prominent benefits for the CNS. The underlying mechanisms and the therapeutic material basis are shown in Figures 2, 3. Our analysis indicates that the main mechanisms involved include the following: antioxidant effects and associated apoptosis; anti-inflammatory effects and related apoptosis; and neurological proliferation, regeneration differentiation, and neuronal synaptic plasticity improvement (Figure 3). Additionally, HPA axis regulation, neurotransmitter release, and receptor improvement (A_2A_R, NMDA-R, GluR, and AMPA-R) contribute to the potential therapeutic profile.

Perhaps the most important value of these findings is to show a scientific basis to promote the development of a drug candidate. Increasing evidence shows that a single molecular target cannot account for all the pathophysiological features in a given neurological disease, and RP seemingly affects many targets relevant to synaptic plasticity, neurogenesis, neuroprotection, and neural transmission, among others. Though molecular and cellular mechanisms of action still need elaboration, it is essential to investigate the neuroprotective effects using pharmacological models with translational value to unravel and assess its therapeutic potential.

## Author Contributions

All authors listed have made a substantial, direct, and intellectual contribution to the work and approved it for publication.

## Funding

This work was supported by the Ministry of Science and Technology of China (2017ZX09301029), the National Key Research and Development Program (No. 2016YFE0131800), and the Development of Animal Model on Human Diseases (2016-I2M-2-006).

## Conflict of Interest

The authors declare that the research was conducted in the absence of any commercial or financial relationships that could be construed as a potential conflict of interest.

## Publisher's Note

All claims expressed in this article are solely those of the authors and do not necessarily represent those of their affiliated organizations, or those of the publisher, the editors and the reviewers. Any product that may be evaluated in this article, or claim that may be made by its manufacturer, is not guaranteed or endorsed by the publisher.
